# Exploring associations between the *FTO* rs9939609 genotype and plasma concentrations of appetite-related hormones in adults with obesity

**DOI:** 10.1371/journal.pone.0312815

**Published:** 2025-01-10

**Authors:** Ann Kristin Hjelle de Soysa, Mette Langaas, Valdemar Grill, Catia Martins, Ingrid Løvold Mostad

**Affiliations:** 1 Outpatient Obesity Clinic, Clinic of Surgery, St. Olavs hospital–Trondheim University Hospital, Trondheim, Norway; 2 Department of Clinical and Molecular Medicine, Faculty of Medicine and Health Sciences, Norwegian University of Science and Technology, Trondheim, Norway; 3 Department of Mathematical Sciences, Faculty of Information Technology and Electrical Engineering, Norwegian University of Science and Technology, Trondheim, Norway; 4 Center for Obesity Research and Innovation (ObeCe), Clinic of Surgery, St. Olavs Hospital–Trondheim University Hospital, Trondheim, Norway; 5 Department of Nutrition and Speech-Language Therapy, Clinic of Rehabilitation, St. Olavs hospital–Trondheim University Hospital, Trondheim, Norway; Sarich Neuroscience Research Institute, AUSTRALIA

## Abstract

Associations between variants in the *FTO* locus and plasma concentrations of appetite related hormones are inconsistent, and might not work in a dose dependent fashion in people with obesity. Moreover, it is relevant to report meal related plasma concentrations of these hormones in persons with obesity given the growing interest in their pharmacological potential in obesity therapy. We find it clinically relevant to examine associations between the SNP rs9939609 genotypes and homeostatic appetite regulation in individuals with BMI ≥35 kg/m^2^. This study explored associations of the rs9939609 genotypes to plasma concentrations of acylated ghrelin, active glucagon-like peptide 1 (GLP-1), and total peptide YY (PYY), and moderating effects of fat mass (FM), in 96 adults (69% female) with BMI ≥35 kg/m^2^, using a cross sectional observation study designed to have 1/3 of participants each with genotypes TT, AT and AA, respectively. Participants were median (25^th^, 75^th^ percentile) 42.5 (32, 50) years of age, weighed 120.9 (109.6, 142.4) kg, and had a BMI of 42.8 (39.5, 46.4) kg/m^2^. Acylated ghrelin, active GLP-1, and total PYY were measured in the fasted state and half-hourly for 2.5h after a standardized meal. We evaluated associations between genotype and appetite hormones in regression analysis controlling for FM and sex. Genotype did not associate with fasting or postprandial (area under curve, AUC) GLP-1 or PYY. Genotype did not associate with fasting acylated ghrelin, but in females with genotype AA, increased FM was associated with higher fasting and postprandial (AUC) acylated ghrelin concentrations relative to genotypes TT (fasting p = 0.025; AUC p = 0.004) and AT (fasting p = 0.002; AUC p < 0.001). This novel finding warrants further investigation.

## Introduction

The prevalence of obesity is still on the rise [1] and causes are multifactorial [2, 3]. Essentially, obesity is the result of a sustained positive energy balance, usually from higher energy intake (EI) [4]. The genotype effect of variants in the fat mass and obesity associated (*FTO*) locus on increased body weight and fat mass (FM) has been studied extensively [5, 6]. Earlier studies found positive associations between *FTO* locus and EI [7], and this has been linked to altered brain food-cue response [8], a preference for more energy dense foods [9], and loss of control over eating [10].

Eating is a highly complex behavior regulated by metabolic, endocrine and neural signals [11] that is typically functionally organized into meals. Initiation of the meal, its size and its termination are controlled by both stimulatory and inhibitory gastro-intestinal signals that communicate with the central nervous system through vagal or non-vagal afferent nerve signaling or via blood circulation [12]. The homeostatic hunger hormone ghrelin rises during fasting and falls after food intake, but is also stimulated in anticipation of eating [13]. Glucagon-like peptide 1 (GLP-1) is released during and after meals and leads to satiation and satiety [14–16], and in recent years GLP-1 analogues are increasingly being used in anti-obesity treatment because of this “acute” eating reducing effect [17]. Peptide YY is mainly involved in post-meal satiety, and stays elevated for several hours after a high protein meal [18, 19].

Obesity (BMI ≥30 kg/m^2^) is associated with attenuated ghrelin concentrations both in the fasted and postprandial state, indicating that people with obesity seem to have weaker satiety signals, but not increased hunger signals [20]. Compared with individuals with normal weight individuals with obesity have been found to have attenuated postprandial secretions of GLP-1 [21, 22] and PYY [19, 23].

A few studies have evaluated associations between the rs9939609 genotype and plasma concentrations of appetite-related hormones (ghrelin, GLP-1, and PYY), and results are inconsistent. In adiposity-matched normal weight young men, those with the AA genotype had an attenuated suppression of acylated ghrelin in the postprandial state relative to TT men (no-risk allele in two copies) [8]. The AA genotype was associated with lower postprandial ghrelin concentrations in women with BMI 40–60 kg/m^2^ [24]. Others [25, 26] did not find associations between *FTO* genotype and fasting and postprandial acyl ghrelin concentrations. A significant *FTO* rs9939609 genotype association with GLP-1 or PYY has not been previously reported [27]. The role of the *FTO* locus in homeostatic appetite regulation in individuals who have already developed obesity, therefore, remains unclear. We [28] have earlier reported that rs9939609 did not associate with fasting and postprandial insulin plasma concentrations.

The present study explored the effect of a standardized meal on ghrelin, GLP-1 and PYY in a sample of adults with obesity, and aimed to examine how FM moderate the association between the rs9939609 genotypes and the plasma concentration of these appetite-related hormones in a sample of men and women with obesity.

## Subjects and methods

### Participants and study design

Eligible participants had been referred to the university hospital’s outpatient obesity clinic and met local criteria for attending the clinic’s information class (age as ≥20 y, and BMI ≥35 kg/m^2^). They were not pregnant, did not have diabetes and had not experienced any incidents of cardiovascular events within the last 4 years. The study was a cross sectional observational metabolic and genetic study.

Recruitment period was between 21^st^ August 2013 and 1^st^ September 2015. Participants were ethnic Norwegians (with a single exception). The sample size was based on power calculation for hepatic insulin sensitivity as described in detail in [28]. The intention was to achieve a sample where an equal number of participants had 0, 1, or 2 copies of the risk allele of the SNP rs9939609 [28]. Of 226 patients consenting to be assessed for eligibility 17 withdrew consent to participate, 56 did not meet inclusion criteria, 33 had HbA1c>5.8% (indicating diabetes); 16 had acute or other medical conditions, 6 lost weight to BMI<35, 1 was pregnant and 7 had begun obesity treatment, had moved, or could not participate due to other reasons). Participant selection was done blinded to participants and investigators. The first 50 participants were included solely according to the inclusion criteria, with no focus on genotype. In a Norwegian population study [29], the frequency of the *FTO* risk (minor) allele rs9939609 among overweight individuals was found to be 0.44, and assuming Hardy-Weinberg equilibrium this gives genotypes probabilities for genotype TT 0.31, AT 0.49, and AA 0.19. To guard against obtaining inadequate numbers of homozygotes for three similarly sized groups, an external controller selected the last 50 participants among the eligible volunteers so that mainly homozygotes were included, excluding 45 patients with genotype AT. One person withdrew consent to be genotyped. Extraction of DNA and genotyping is previously described [28]. The study was approved by the Norwegian Regional Committees for Medical and Health Research (2013/642/REK midt) and was conducted according to the guidelines of the Declaration of Helsinki. All volunteers provided written informed consent to participate.

### Meal test

Acylated ghrelin, active GLP-1, and total PYY were measured in the fasted state and after a standardized meal. Participants were instructed not to exercise (allowed to walk at a leisurely pace) or use alcohol in the 24h preceding the meal test. Participants arrived at 08:15h in the morning after 10h of overnight fasting, including a temporary discontinuation of medications and tobacco-products. There was no assessment of food preferences, but participants were asked if they preferred milk or drinking-yoghurt with their standardized test meal. None had allergies that we had to consider. They then consumed a standardized meal of whole grain bread, butter, cheese, jam, orange juice, and either milk or sweetened yoghurt drink (600 kcal, 48% of energy (E%) as carbohydrates (CHO), 17 E% protein and 35 E% fat) within 15 minutes [30]. The meal weight was 500 g for those choosing milk and 425 g for those choosing yoghurt instead of milk. Blood samples for the analysis of acylated ghrelin, active GLP-1, and total PYY were collected into EDTA-coated tubes in the fasted state and repeatedly every 30 minutes for 2.5h after the meal. Around 1 mL of full blood was then transferred into a micro tube and 20 μl mixture of inhibitors (10 μl of Pefabloc (Roche Diagnostic, Mannheim, Germany) + 10 μl DPP-IV (Merck Millipore, Germany)) added. The micro tubes were immediately centrifuged (2110 RCF, 10 min, 18°C), and the plasma was kept at -80°C pending analyses. There was no refreezing of thawed samples. All samples from each participant were analyzed in duplicates in the same assay run. Plasma acylated ghrelin, active GLP-1, and total PYY were measured using a Human Metabolic Hormone Magnetic Bead Panel multikit (LINCOplex Kit, Millipore, Merck KGaA, Darmstadt, Germany). Every sample was given a unique and random barcode and registered in Biobank1®. The same technician analyzed all the samples. Mean intra- and inter-assay CV for the quality controls in our analyses were 4.2% and 4.6% for ghrelin, 4.3% and 5.0% for GLP-1, and 5.7% and 3.9% for PYY. We used only results for which we had detectable values (i.e. no imputation method was used for undetectable values). Areas under curve (AUC) for plasma concentrations of appetite hormones from fasting value (0 minutes), 30, 60, 90, 120 and 150 minutes after the standardized meal, were calculated using the trapezoid rule. After the meal test the participants gave a 24 h diet interview pertaining to the intake in the 24 h preceding the meal test [31]. Participants also filled in a questionnaire that included questions on habitual weekly meal frequencies (breakfast, lunch, warm dinner meal, evening meal, night food) [32].

### Anthropometry and body composition

Measures of weight, height, and body composition were done following procedures reported elsewhere [28]. Fat mass (FM) and fat free mass (FFM) were measured using dual energy x-ray absorptiometry (DXA) (Holigic, Inc., Apex Software, Bedford, MA, USA). The DXA scan included head, trunk and legs, but not arms because the size of the bench was too small. Compartmentalization of the body without the arms was performed in a standardized way to avoid in-between subject differences due to inaccurate measurements.

### Statistics

We performed statistical analyses with the STATA package, version 17 (StataCorp, College Station, Texas, USA) [33] and in R [34], and made figures in R and Excel. The distribution of the background variables was inspected by histograms, and normality examined with the Shapiro-Wilks test. We present medians, 25th and 75th percentile values. For one-way analysis of variance of the participant characteristics, we used the Kruskal-Wallis method, robust regression (“rreg**”** in Stata) [35], and Fisher’s exact test for categorical outcomes (i.e. sex). Associations between genotype and appetite related hormones were analyzed with multiple linear regression. Because the *FTO* locus is associated with increased FM, and FM is known to affect fasting ghrelin [36] and GLP-1 concentrations [37], we controlled for FM in the additive model, and given that the effect of genotype on ghrelin might be influenced by FM, we also included this variable as an interaction term (genotype*FM). This interaction term allowed us to examine the effect of genotype on ghrelin conditional on FM level. To account for an influence of biological sex, we additionally included sex as a covariate in a larger model. Finally, the effect of genotype conditional on FM for acylated ghrelin was tested separately for men and women.

We inspected the residuals from the regression analyses with histograms, QQ-plots and the Shapiro-Wilk normality test. Fasting and AUC for ghrelin and GLP-1 were natural log-transformed in regression analyses. For all regression analyses genotype had codominant coding, that is, a factor with three levels denoted by the number of risk allele copies present, thus 0, 1, or 2 for genotypes TT, AT, and AA, respectively. Due to the multiple testing burden, we opted to consider a p-value ≤ 0.01 as a significant association in this exploratory study. We calculated a power of 54% of the observed effect size (change in R^2^, partial eta^2^) of AUC for ghrelin, with FM as a covariate in addition to genotype, retrospectively using G*Power, version 3.1.9.7 [38].

## Results

### Participants

Ninety-six participants (69% females) completed the meal test for investigating postprandial appetite hormone concentrations. The genotype groups were similar in the number of participants who completed the test. There were no significant differences in participant characteristics between genotype groups (**Table 1**). Energy intakes did not differ between genotype groups (p = 0.424). Habitual meal frequencies and breakfast meals did not differ between genotype groups [32]. Males had higher weight, FFM and energy intakes than females (Table 1, footnotes). Fat mass (FM) did not differ between males and females (47 (38, 53) vs. 45 (40, 52) kg), respectively, (p = 0.879).

**Table 1:** Characteristics of study population, by genotype and for the whole sample.

	All	TT	AT	AA
	*n* = 96	*n* = 32	*n* = 30	*n* = 34
Males/females, n	66/30	26/6	18/12	22/12
Age, year	42.5	39.5	44	44
	(32, 50)	(30.5, 46.5)	(37, 51)	(31, 53)
Weight, kg	120.9^a^	119.8	120.9	127.1
	(109.6, 142.4)	(106.0, 141.3)	(109.5, 142.3)	(115.4, 142.4)
BMI, kg/m^2^	42.8	43.1	40.8	43.1
	(39.5, 46.4)	(38.2, 46.6)	(37.8, 45.3)	(40.5, 47.4)
FM^1^, kg	45.7	48.3	41.4	46.3
	(38.8, 53.5)	(40.0, 55.8)	(37.3, 51.6)	(39.9, 52.3)
FFM^1^, kg	64.3^b^	63.4	68.0	63.7
	(59.3, 78.3)	(59.0, 72.0)	(59.4, 80.5)	(58.4, 76.7)
Energy intake, kcal	2098^c^	2050	2145	2037
	(1530, 2546)	(1363, 2438)	(1831, 2582)	(1431, 2540)

Data are Median (25^th^, 75^th^ percentile). BMI: body mass index; FM: fat mass; FFM: fat free mass.

^1^ From DXA measurement, measurements are without arms, *n* = 95.

^a^ Body weight: males 147.6 (129.4, 155.6) kg vs. females 117.8 (105.2, 128.4) kg, p < 0.001.

^b^ FM: males 83.3 (78.3, 90.1) kg vs. females 60.8 (57.2, 64.6) kg, p < 0.001.

^c^ Males 2439 (1951, 2664) kcal vs. females 1928 (1431, 2372) kcal, p = 0.019.

### Appetite hormones

**Table 2** shows median (25^th^, 75^th^ percentiles) values for fasting and AUC for the hormones, by genotype groups. There were no differences in fasting concentrations of ghrelin, GLP-1 or PYY between genotypes (Table 2). Median (25^th^, 75^th^ percentiles) concentrations of acylated ghrelin, active GLP-1 and total PYY during the meal-test time points for males and females are depicted in **S1 Fig** in Supporting information.

**Table 2:** Appetite related hormones in fasted and postprandial state, for all participants and by genotype.

	All	TT	AT	AA
**Acylated ghrelin**	*n* = 96	*n* = 32	*n* = 30	*n* = 34
Fasting, pmol/L	20.6	22.0^1^	17.5^1^	20.3^1^
	(13.1, 31.7)	(13.6, 37.3)	(12.2, 28.8)	(13.4, 34.3)
AUC, pmol/L*min	2128	2618	1917	2068
	(1527, 3075)	(1698, 3695)	(1344, 2972)	(1372, 3086)
**Active GLP-1**	*n* = 94	*n* = 32	*n* = 29	*n* = 33
Fasting, pmol/L	0.8	0.8^2^	0.8^2^	0.8^2^
	(0.8, 0.8)	(0. 8, 0.8)	(0. 8, 0.9)	(0.8, 0.8)
AUC, pmol/L*min	327	361	308	331
	(238, 531)	(224, 486)	(244, 592)	(220, 508)
**Total PYY**	*n* = 29	*n* = 8	*n* = 9	*n* = 12
Fasting, pmol/L	10.2	9.8^3^	13.4^3^	8.9^3^
	(6.6, 17.2)	(5.9, 24.9)	(7.2, 15.2)	(6.6, 16.9)
AUC, pmol/L*min	2060	2246	2062	1972
	(1505, 2808)	(1387, 3785)	(1871, 2167)	(1447, 2683)

Data are median (25^th^, 75^th^ percentile). AUC, total area under curve; GLP-1, glucagon-like peptide-1; PYY, peptide YY.

Conversions from metric to SI units were done as follows: ghrelin pg/mL x 0.3 = pmol/L, GLP-1 pg/mL x 0.33 = pmol/L, and PYY pg/mL x 0.25 = pmol/L.

^1^ p values 0.082 (AT vs. TT), 0.262 (AA vs.TT), 0.489 (AA vs. AT)

^2^p values 0.241 (AT vs. TT), 0.739 (AA vs.TT), 0.384 (AA vs. AT)

^3^ p values 0.700 (AT vs. TT), 0.685 (AA vs.TT), 0.405 (AA vs. AT)

#### Ghrelin

**Fig 1:**
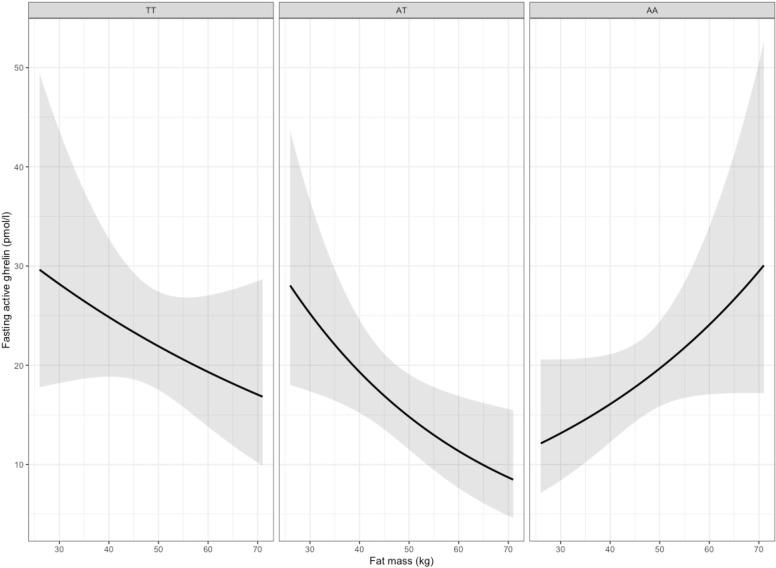
Median acylated ghrelin over time during meal test for each genotype group. Solid line shows median and light grey area shows the area from the 25^th^ (Q1) to the 75^th^ percentile (Q3). For ghrelin, pg/mL x 0.3 gives ghrelin value in pmol/L.

There was a significant effect of time on ghrelin (i.e. significant decrease from fasting level) (P<0.001). **Fig 1** shows median acylated ghrelin over time for each genotype group. Controlling for FM and sex we found a genotype association with fasting and AUC acylated ghrelin, and a FM*genotype interaction effect on ghrelin (**S1–S4 Tables**). In sex-stratified analyses we observed this conditional effect (FM*genotype) for women on fasting acylated ghrelin (p = 0.025 for AA vs. TT, and p = 0.002 for AA vs. AT) and AUC (p = 0.004 for AA vs. TT, and p < 0.001 for AA vs. AT) (**S5 and S6 Tables**), but not for men (**S7 and S8 Tables**).

**Fig 2:**
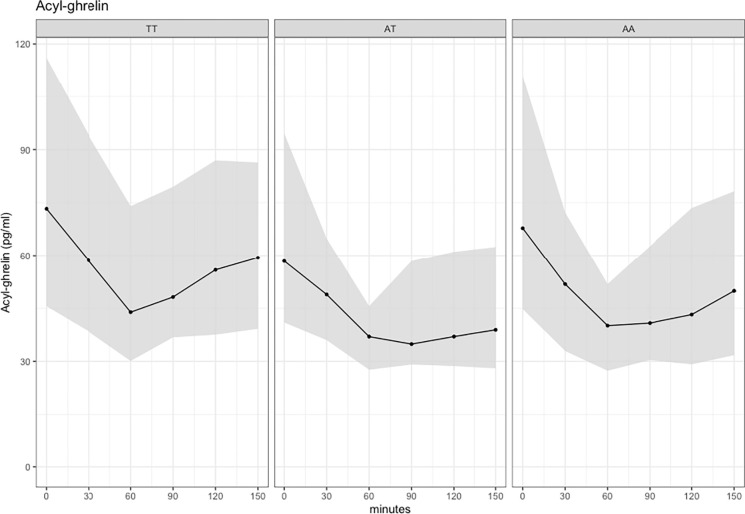
Conditional effect of fat mass (FM) on fasting acylated ghrelin for each genotype group. The solid line is the fitted regression model (model with interaction effect of FM*genotype, fitted with log-acylated ghrelin as response) and the shaded area is the 95% confidence intervals for the regression line presented on the original scale for acylated ghrelin. *P* value is 0.0105 for the differences between the slopes (the genotype*FM interaction effect). For ghrelin, pmol/L x 3.371 gives ghrelin value in pg/mL.

When we included the interaction term genotype*FM, each genotype had its own slope in FM (**Fig 2**) (p = 0.0105 for the differences between the slopes). For genotype AA, increasing FM was associated with higher fasting acylated ghrelin concentration. In this fitted model, when FM increased from 26 to 71 kg, predicted mean fasting acylated ghrelin increased from 12.1 to 30.1 pmol/L in the AA group and decreased from 29.6 to 16.9 pmol/L in the TT group (p = 0.037) and from 28.1 to 8.5 pmol/L in the AT group (p = 0.003).

#### GLP-1 and PYY

There was a significant postprandial increase in active GLP-1 (P<0.001). We did not find associations between genotype and active GLP-1 or total PYY. Total PYY was detectable and measurable in29 participants, that is, 33% of men and 29% of women. It was measurable in 25% of TT, 30% of AT, and 35% of the AA participants.

## Discussion

This study was set up to explore a possible impact of an obesity-related variant in the *FTO* locus, rs9939609, on homeostatic appetite control. In contrast to previous studies, our population had median BMI obesity class III and was selected for equal representation of the different genotypes of rs9939609. Similar to previous research in individuals with obesity, appetite hormone levels were lower at baseline (fasting) and postprandial responses tended to be blunted [20, 21, 23]. Genotype affected the important appetite-regulating hormone ghrelin, and in some aspects the regulation was different for participants with the double risk variant AA.

Our study found associations between genotype and ghrelin in additive and interaction analyses, i.e. fasting concentrations and postprandial response (AUC) were lower in participants with the risk allele (AA or AT relative to TT). This is somehow in line with results from Karra *et al*. [8], reporting attenuated postprandial ghrelin response among young, normal weight men with genotype AA relative to TT. Benedict *et al*. [39] found a positive association between fasting total ghrelin and *FTO* genotype risk. Our result is in contrast with Danaher *et al*. [25] and Goltz *et al*. [26] who did not find any genotype effect on pre- and postprandial levels of appetite-related hormones, even after adjusting for BMI and sex [25], or additionally adjusting for other measures of body composition and age [26]. Melhorn *et al*. [27] using a recessive model (TT+ AT versus AA) in a sample that was enriched with individuals with obesity, and Magno *et al*. [24], studying women with BMI between 40 and 60 kg/m^2^, found that the double risk allele (AA) was associated with a greater drop in postprandial ghrelin relative to the other groups. We found a significant decrease in plasma concentrations of acylated ghrelin from the fasted to the postprandial state. This result differs from a study that used the same meal test and the same biochemical procedures [22]. Differences in experimental procedures, study populations, as well as statistical methods and models may explain some of the discrepancies seen among studies. Dorling *et al*. [40] showed that physical activity affected appetite through hydrolyzation of acyl ghrelin to deacyl ghrelin, decreasing acyl ghrelin concentration. Acylated ghrelin is the ghrelin form that is able to cross the blood brain barrier and act at the level of the brain to module appetite [41, 42]. Even though deacylated ghrelin may exert other functions, we feel that for the aim of this study acylated ghrelin was the most relevant ghrelin form to measure.

A novel finding of ours was a strong and positive association between FM size and plasma acylated ghrelin concentration in the AA genotype. For genotype AA, our statistical model showed a positive association between FM size and fasting ghrelin and AUC for ghrelin, whereas for genotypes AT and TT, there was an inverse association between FM size and ghrelin concentrations, as would be expected since FM is inversely associated with fasting acylated ghrelin. Ghrelin, in addition to playing a role in the homeostatic appetite regulation and feeding [27], which was the objective of our study, is implicated in hedonic and motivational feeding [43]. Ovarian sex hormones are also implicated in regulation of food intake and energy balance [44], and importantly, an interaction between ghrelin and sex hormones in females exists, but this is a complex and understudied area [44]. Variants in the *FTO* locus are expressed in brain regions that drive food intake [45], and have been associated with reward processing and motivation to eat [8], binge eating [46, 47], and reduced satiety [27]. Our observation of a positive association between FM and ghrelin in AA-participants could be the combined effects that the risk allele exert on these brain circuits (i.e. increase ghrelin), as well as an unknown impact of any sex hormones. In other words, what was observed in our model could be the long term result of the neural response of the AA-allele on drive to eat (e.g. increased FM as a result of increased food intake). Further studies are warranted to confirm and validate our results.

In sex-stratified analyses we observed that the conditional genotype effect was pronounced in women, but it could not be confirmed in the smaller sample of men. This highlights the need to conduct tests in large enough samples of males and females. Potential metabolic differences between the two sexes need further clarifications in the future.

We found a significant increase in plasma concentrations of active GLP-1 from the fasted to the postprandial stage for the study population, but in line with Melhorn *et al* [27], we did not find associations between rs9939609 genotype and active GLP-1.

In accordance with others [22, 48], we too found that fasting PYY concentrations were low and postprandial PYY responses were attenuated in our sample of individuals with severe obesity (median BMI 42.8). Moreover, only 30% of our participants had detectable PYY concentrations and this suggests that their fasting concentrations were below 3.8 pmol/L (15.1 pg/mL) which was our detection limit. Obviously our data on PYY should therefore be interpreted with due caution.

Our study has several strengths. First, few studies have reported appetite related hormones in individuals with BMI obesity classes II and III. Mapping both fasting and postprandial plasma concentrations of gastrointestinal hormones in a large sample of individuals with obesity such as ours, is therefore important in its own right. Second, the hormone analyses had low intra- and inter-variability, something which indicates good reliability. Third, controlling as here for FM obtained from DXA measurements rather than BMI or body weight in the statistical analyses seems to be the most valid approach. Finally, a major strength is that we report our data for each genotype of the rs9939609.

The study has limitations. It was exploratory and not powered specifically for analyzing genotype effect on appetite related hormones. The meal prior to the 10 h fasting before the meal test was not standardized and could have influenced fasting concentration of GLP-1 and PYY [49]. There was no control of habitual breakfast consumption and this could have influenced morning appetite hormones. Another limitation is that the hormones were measured using a multiplex assay rather than assays optimized for each individual hormone, possibly giving less precise data than otherwise achievable, data on PYY being a case in point. Finally, we did not register the ovarian hormone status in the female participants.

We believe that by gaining greater insight into the associations of the rs9939609 SNP risk allele on appetite in this population will equip clinicians with more knowledge in dealing with patients directly, and might add to the body of evidence on future strategies for preventing and treating obesity in general. Our data show large inter-individual variations in both fasting and postprandial plasma concentrations of appetite-related hormones in individuals with obesity. Our results taken in the context of increasing focus on GLP-1 analogues and other gut hormones in pharmacological obesity treatment [16, 17], adds further value towards understanding the complexities of appetite regulation in this population.

In summary, we found that in a population of men and women with obesity, the effect of genotype AA on plasma ghrelin concentration was conditional on FM size. Among those with two copies of the rs9939609 risk allele, greater FM was associated with a higher plasma ghrelin concentration.

## Supporting information

S1 Fig Median appetite hormones over time (min) during meal test for males on left side (panel A) and females on right side (panel B).
(PDF)

S1 Table Effect of fat mass (FM) and genotype on fasting ghrelin concentration. (PDF)

S2 Table Effect of fat mass (FM) and genotype on ghrelin AUC. (PDF)

S3 Table Effect of biological sex, fat mass (FM) and genotype on fasting ghrelin concentrations. (PDF)

S4 Table Effect of biological sex, fat mass (FM) and genotype on ghrelin AUC. (PDF)

S5 Table Effect of fat mass (FM) and genotype on fasting ghrelin concentrations in females (n = 65). (PDF)

S6 Table Effect of fat mass (FM) and genotype on ghrelin AUC in females (n = 65). (PDF)

S7 Table Effect of fat mass (FM) and genotype on fasting ghrelin concentrations in males (n = 30). (PDF)

S8 Table Effect of fat mass (FM) and genotype on ghrelin AUC in males (n = 30). (PDF)
