# Supplementary material for: Exploring associations between the FTO rs9939609 genotype and plasma concentrations of appetite-related hormones in adults with obesity
Source: PLoS One. 2025 Jan 10;20(1):e0312815. doi: 10.1371/journal.pone.0312815 (PMC11723609; doi:10.1371/journal.pone.0312815)
Supplement: S1 Fig — Median appetite hormones over time (min) during meal test for males on left side (panel A) and females on right side (panel B). (PDF) [file pone.0312815.s001.pdf]

Supporting information

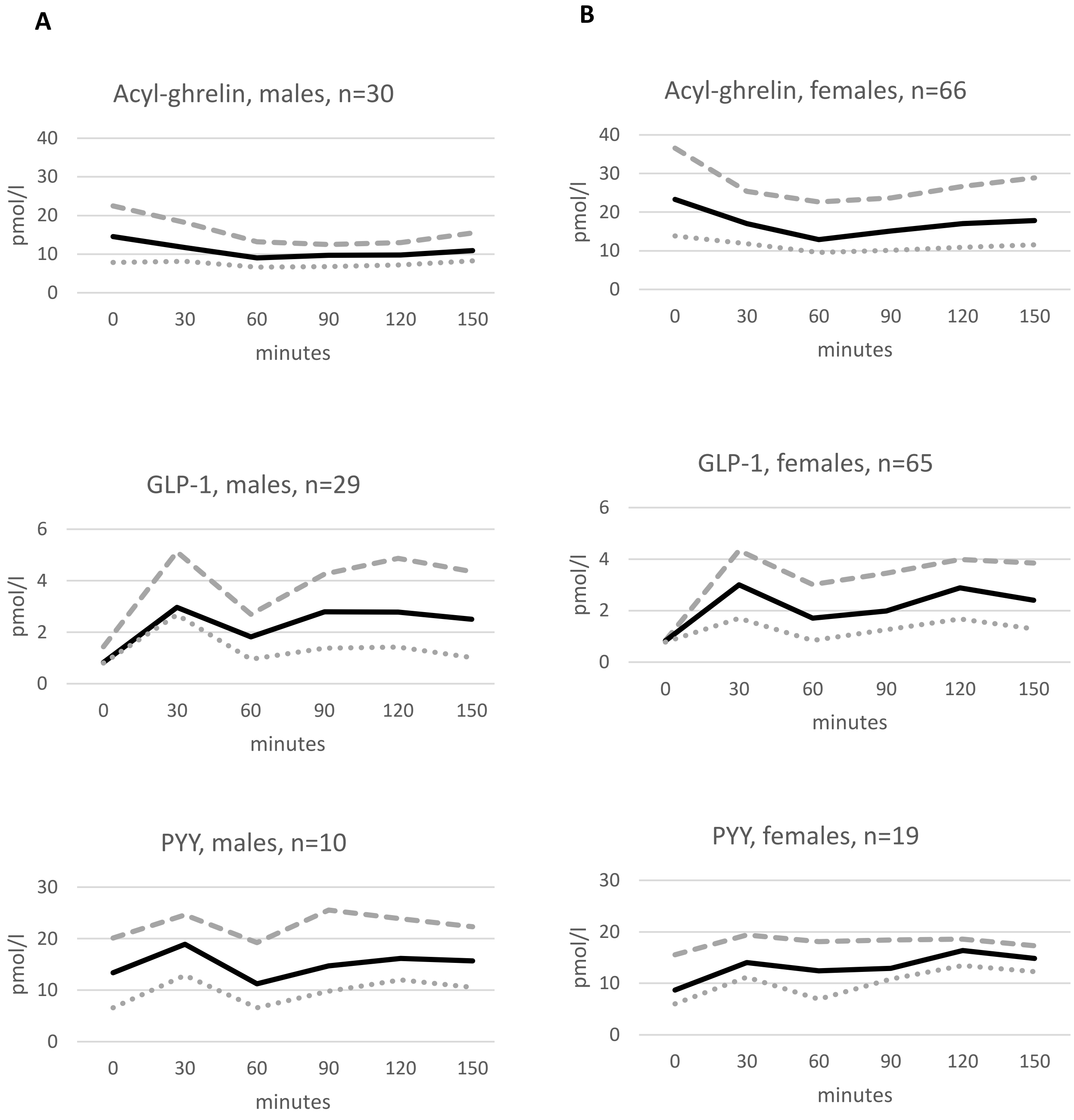

S1 Fig. Median appetite hormones over time (min) during meal test for males on left side (panel A) and females on right side (panel B).

Solid black line shows median, grey dotted lines show the 25th and 75th percentile.
Conversions from metric to SI units were done as follows: ghrelin pg/ml x 0.3 = pmol/l, GLP-1 pg/ml x 0.33 = pmol/l, and PYY pg/ml x 0.25 = pmol/l

---

Exploring associations between the FTO rs9939609 genotype and plasma concentrations of appetite-related hormones in adults with obesity.

Ann Kristin Hjelle de Soysa, Mette Langaas, Valdemar Grill, Catia Martins, Ingrid Løvold Mostad
