# Supplementary material for: Exploring associations between the FTO rs9939609 genotype and plasma concentrations of appetite-related hormones in adults with obesity
Source: PLoS One. 2025 Jan 10;20(1):e0312815. doi: 10.1371/journal.pone.0312815 (PMC11723609; doi:10.1371/journal.pone.0312815)
Supplement: S1 Table — (PDF) [file pone.0312815.s002.pdf]

S1 Table. Effect of fat mass (FM) and genotype on fasting ghrelin concentration.

Robust regression FM + genotype + genotype*FM, pairwise comparisons of marginal linear predictions

Acylated ghrelin, fasting	Coefficient	Std. error	P-value	95% Conf. interval
FM	-.012	.011	0.281	-.033, .0097
Genotype				
1 vs 0	.355	.719	0.623	-1.073, 1.783
2 vs 0	-1.950	.767	0.013	-3.474, -.426
2 vs 1	-2.305	.739	0.002	-3.774, -.836
Genotype*FM				
1 vs 0	-.014	.015	0.346	-.045, .016
2 vs 0	.038	.016	0.019	.006, .069
2 vs 1	.052	.016	0.001	.021, .083
_cons	4.894	.528	0.000	3.844, 5.944

Number of obs	=	95
F(5, 89)	=	2.91
Prob > F	=	0.0177

Dependent variable acylated ghrelin concentration (pg/ml) is natural log-transformed in analyses; FM, fat mass (kg) obtained from DXA measurement, measurements are without arms; Genotype, 0=TT, 1=AT, and 2=AA; AUC, total area under curve.
