# Supplementary material for: Exploring associations between the FTO rs9939609 genotype and plasma concentrations of appetite-related hormones in adults with obesity
Source: PLoS One. 2025 Jan 10;20(1):e0312815. doi: 10.1371/journal.pone.0312815 (PMC11723609; doi:10.1371/journal.pone.0312815)
Supplement: S2 Table — (PDF) [file pone.0312815.s003.pdf]

S2 Table. Effect of fat mass (FM) and genotype on ghrelin AUC.

Regression FM + genotype + genotype*FM, pairwise comparisons of marginal linear predictions

Acylated ghrelin, AUC	Coefficient	Std. error	P-value	95% Conf. interval
FM	-.008	.009	0.371	-.027, .010
Genotype				
1 vs 0	.125	.618	0.841	-1.104, 1.353
2 vs 0	-2.010	.660	0.003	-3.321, -.699
2 vs 1	-2.134	.636	0.001	-3.398, -.871
Genotype*FM				
1 vs 0	-.011	.013	0.405	-.037, .015
2 vs 0	.0378	.013	0.006	.011, .065
2 vs 1	.049	.013	0.000	.022, .075
_cons	9.494	.454	0.000	8.591, 10.397

Number of obs	=	95	R-squared	=	0.1833
F(5, 89)	=	3.99	Adj. R-squared	=	0.1374
Prob > F	=	0.0026	Root MSE	=	.53826

Dependent variable acylated ghrelin concentration (pg/ml) is natural log-transformed in analyses; FM, fat mass (kg) obtained from DXA measurement, measurements are without arms; Genotype, 0=TT, 1=AT, and 2=AA; AUC, total area under curve.
