# Supplementary material for: Exploring associations between the FTO rs9939609 genotype and plasma concentrations of appetite-related hormones in adults with obesity
Source: PLoS One. 2025 Jan 10;20(1):e0312815. doi: 10.1371/journal.pone.0312815 (PMC11723609; doi:10.1371/journal.pone.0312815)
Supplement: S3 Table — (PDF) [file pone.0312815.s004.pdf]

S3 Table. Effect of biological sex, fat mass (FM) and genotype on fasting ghrelin concentrations.

Robust regression sex + FM + genotype + genotype*FM, pairwise comparisons of marginal linear predictions

Acylated ghrelin, fasting	Coefficient	Std. error	P-value	95% Conf. interval
Sex	.521	.136	0.000	.250, .792
FM	-.007	.010	0.528	-.027, .014
Genotype				
1 vs 0	.858	.698	0.222	-.529, 2.245
2 vs 0	-1.334	.744	0.076	-2.812, .144
2 vs 1	-2.192	.705	0.003	-3.592, -.792
Genotype*FM				
1 vs 0	-.024	.015	0.109	-.053, .005
2 vs 0	.026	.015	0.093	-.004, .056
2 vs 1	.049	.015	0.001	.020, .079
_cons	4.239	.533	0.000	3.181, 5.298

Number of obs	=	95
F(6, 88)	=	5.11
Prob > F	=	0.0002

Dependent variable acylated ghrelin concentration (pg/ml) is natural log-transformed in analyses; FM, fat mass (kg) obtained from DXA measurement, measurements are without arms; Genotype, 0=TT, 1=AT, and 2=AA; AUC, total area under curve.
