# Supplementary material for: Exploring associations between the FTO rs9939609 genotype and plasma concentrations of appetite-related hormones in adults with obesity
Source: PLoS One. 2025 Jan 10;20(1):e0312815. doi: 10.1371/journal.pone.0312815 (PMC11723609; doi:10.1371/journal.pone.0312815)
Supplement: S4 Table — (PDF) [file pone.0312815.s005.pdf]

S4 Table. Effect of biological sex, fat mass (FM) and genotype on ghrelin AUC.

Regression sex + FM + genotype + genotype*FM, pairwise comparisons of marginal linear predictions

Acylated ghrelin, AUC	Coefficient	Std. error	P-value	95% Conf. interval
Sex	.4465251	.1141916	0.000	.220, .673
FM	-.0040198	.0086681	0.644	-.021, .013
Genotype				
1 vs 0	.5581691	.5844169	0.342	-.603, 1.720
2 vs 0	-1.56485	.6227981	0.014	-2.803, -.327
2 vs 1	-2.123019	.5901261	0.001	-3.296, -.950
Genotype*FM				
1 vs 0	-.0182801	.012268	0.140	-.043, .006
2 vs 0	.029979	.0126873	0.020	.005, .055
2 vs 1	.0482591	.0124945	0.000	.023, .073
_cons	8.927785	.4460573	0.000	8.041, 9.814

Number of obs	=	95	R-squared	=	0.3042
F(6, 88)	=	6.41	Adj. R-squared	=	0.2567
Prob > F	=	0.0000	Root MSE	=	.49964

Dependent variable acylated ghrelin concentration (pg/ml) is natural log-transformed in analyses; FM, fat mass (kg) obtained from DXA measurement, measurements are without arms; Genotype, 0=TT, 1=AT, and 2=AA; AUC, total area under curve.

Exploring associations between the FTO rs9939609 genotype and plasma concentrations of appetite-related hormones in adults with obesity.

Ann Kristin Hjelle de Soysa, Mette Langaas, Valdemar Grill, Catia Martins, Ingrid Løvold Mostad
