# Supplementary material for: Exploring associations between the FTO rs9939609 genotype and plasma concentrations of appetite-related hormones in adults with obesity
Source: PLoS One. 2025 Jan 10;20(1):e0312815. doi: 10.1371/journal.pone.0312815 (PMC11723609; doi:10.1371/journal.pone.0312815)
Supplement: S5 Table — (PDF) [file pone.0312815.s006.pdf]

S5 Table. Effect of fat mass (FM) and genotype on fasting ghrelin concentrations in females (n=65).
Robust regression FM + genotype + genotype*FM, pairwise comparisons of marginal linear predictions in females

**Acylated ghrelin, fasting**	**Coefficient**	**Std. error**	**P-value**	**95% Conf. interval**
FM	-.012	.010	0.248	-.033, .009
Genotype				
1 vs 0	.696	.733	0.346	-.770, 2.162
2 vs 0	-1.670	.779	0.036	-3.229, -.111
2 vs 1	-2.366	.810	0.005	-3.988, -.744
Genotype*FM				
1 vs 0	-.018	.015	0.252	-.049, .013
2 vs 0	.037	.016	0.025	.005, .068
2 vs 1	.055	.017	0.002	.021, .088
_cons	4.925	.494	0.000	3.937, 5.912

Number of obs	=	65
F(5, 59)	=	2.70
Prob > F	=	0.0291

Dependent variable acylated ghrelin concentration (pg/ml) is natural log-transformed in analyses; FM, fat mass (kg) obtained from DXA measurement, measurements are without arms; Genotype, 0=TT, 1=AT, and 2=AA; AUC, total area under curve.
