# Supplementary material for: Exploring associations between the FTO rs9939609 genotype and plasma concentrations of appetite-related hormones in adults with obesity
Source: PLoS One. 2025 Jan 10;20(1):e0312815. doi: 10.1371/journal.pone.0312815 (PMC11723609; doi:10.1371/journal.pone.0312815)
Supplement: S6 Table — (PDF) [file pone.0312815.s007.pdf]

S6 Table. Effect of fat mass (FM) and genotype on ghrelin AUC in females (n=65).

Regression FM + genotype + genotype*FM, pairwise comparisons of marginal linear predictions in females

**Acylated ghrelin, AUC**	**Coefficient**	**Std. error**	**P-value**	**95% Conf. interval**
FM	-.0092363	.0093292	0.326	-.028, .009
Genotype				
1 vs 0	.4310437	.6623437	0.518	-.894, 1.756
2 vs 0	-2.079246	.7042702	0.005	-3.488, -.670
2 vs 1	-2.51029	.7325442	0.001	-3.976, -1.04
Genotype*FM				
1 vs 0	-.0145106	.0140037	0.304	-.043, .014
2 vs 0	.04346	.0143818	0.004	.015, .072
2 vs 1	.0579706	.0151285	0.000	.028, .088
_cons	9.558787	.446136	0.000	8.666, 10.452

Number of obs	=	65	R-squared	=	0.2416
F(5, 59)	=	3.76	Adj. R-squared	=	0.1774
Prob > F	=	0.0051	Root MSE	=	.49295

Dependent variable acylated ghrelin concentration (pg/ml) is natural log-transformed in analyses; FM, fat mass (kg) obtained from DXA measurement, measurements are without arms; Genotype, 0=TT, 1=AT, and 2=AA; AUC, total area under curve.
