# Supplementary material for: Exploring associations between the FTO rs9939609 genotype and plasma concentrations of appetite-related hormones in adults with obesity
Source: PLoS One. 2025 Jan 10;20(1):e0312815. doi: 10.1371/journal.pone.0312815 (PMC11723609; doi:10.1371/journal.pone.0312815)
Supplement: S7 Table — (PDF) [file pone.0312815.s008.pdf]

S7 Table. Effect of fat mass (FM) and genotype on fasting ghrelin concentrations in males (n=30). Robust regression FM + genotype + genotype*FM, pairwise comparisons of marginal linear predictions in males

**Acylated ghrelin, fasting**	**Coefficient**	**Std. error**	**P-value**	**95% Conf. interval**
FM	-.004	.039	0.917	-.085, .077
Genotype				
1 vs 0	.700	2.33	0.767	-4.115, 5.515
2 vs 0	-.610	2.417	0.803	-5.600, 4.379
2 vs 1	-1.310	1.519	0.397	-4.445, 1.824
Genotype*FM				
1 vs 0	-.028	.045	0.535	-.122, .065
2 vs 0	.002	.047	0.964	-.098, .094
2 vs 1	.026	.034	0.441	-.043, .096
_cons	4.477	2.119	0.045	.104, 8.850

Number of obs	=	30
F(5, 24)	=	1.09
Prob > F	=	0.3929

Dependent variable acylated ghrelin concentration (pg/ml) is natural log-transformed in analyses; FM, fat mass (kg) obtained from DXA measurement, measurements are without arms; Genotype, 0=TT, 1=AT, and 2=AA; AUC, total area under curve.
