# Supplementary material for: Exploring associations between the FTO rs9939609 genotype and plasma concentrations of appetite-related hormones in adults with obesity
Source: PLoS One. 2025 Jan 10;20(1):e0312815. doi: 10.1371/journal.pone.0312815 (PMC11723609; doi:10.1371/journal.pone.0312815)
Supplement: S8 Table — (PDF) [file pone.0312815.s009.pdf]

S8 Table. Effect of fat mass (FM) and genotype on ghrelin AUC in males (n=30).

Regression FM + genotype + genotype*FM, pairwise comparisons of marginal linear predictions in males

**Acylated ghrelin, AUC**	**Coefficient**	**Std. error**	**P-value**	**95% Conf. interval**
FM	.00873	.026218	0.742	-.045, .063
Genotype				
1 vs 0	.865158	1.557096	0.584	-2.349, 4.079
2 vs 0	-.1675907	1.613507	0.918	-3.498, 3.163
2 vs 1	-1.032749	1.013767	0.318	-3.125, 1.060
Genotype*FM				
1 vs 0	-.0276898	.0302146	0.369	-.090, .035
2 vs 0	-.0067172	.0310613	0.831	-.071, .057
2 vs 1	.0209726	.0224268	0.359	-.025, .067
_cons	8.492329	1.414254	0.000	5.573, 11.411

Number of obs	=	30	R-squared	=	0.2114
F(5, 24)	=	1.29	Adj. R-squared	=	0.0471
Prob > F	=	0.3023	Root MSE	=	.50131

Dependent variable acylated ghrelin concentration (pg/ml) is natural log-transformed in analyses; FM, fat mass (kg) obtained from DXA measurement, measurements are without arms; Genotype, 0=TT, 1=AT, and 2=AA; AUC, total area under curve.
